# Oleoyl-Lysophosphatidylcholine Limits Endothelial Nitric Oxide Bioavailability by Induction of Reactive Oxygen Species

**DOI:** 10.1371/journal.pone.0113443

**Published:** 2014-11-24

**Authors:** Andrijana Kozina, Stefan Opresnik, Michael Sze Ka Wong, Seth Hallström, Wolfgang F. Graier, Roland Malli, Katrin Schröder, Kurt Schmidt, Saša Frank

**Affiliations:** 1 Institute of Molecular Biology and Biochemistry, Center of Molecular Medicine, Medical University of Graz, 8010 Graz, Austria; 2 Institute of Cardiovascular Physiology, Faculty of Medicine, Goethe-University Frankfurt, 60590 Frankfurt, Germany; 3 Institute of Physiological Chemistry, Center of Physiological Medicine, Medical University of Graz, 8010 Graz, Austria; 4 Department of Pharmacology and Toxicology, Karl-Franzens-University of Graz, 8010 Graz, Austria; Max-Delbrück Center for Molecular Medicine (MDC), Germany

## Abstract

Previously we reported modulation of endothelial prostacyclin and interleukin-8 production, cyclooxygenase-2 expression and vasorelaxation by oleoyl- lysophosphatidylcholine (LPC 18:1). In the present study, we examined the impact of this LPC on nitric oxide (NO) bioavailability in vascular endothelial EA.hy926 cells. Basal NO formation in these cells was decreased by LPC 18:1. This was accompanied with a partial disruption of the active endothelial nitric oxide synthase (eNOS)- dimer, leading to eNOS uncoupling and increased formation of reactive oxygen species (ROS). The LPC 18:1-induced ROS formation was attenuated by the superoxide scavenger Tiron, as well as by the pharmacological inhibitors of eNOS, NADPH oxidases, flavin-containing enzymes and superoxide dismutase (SOD). Intracellular ROS-formation was most prominent in mitochondria, less pronounced in cytosol and undetectable in endoplasmic reticulum. Importantly, Tiron completely prevented the LPC 18:1-induced decrease in NO bioavailability in EA.hy926 cells. The importance of the discovered findings for more in vivo like situations was analyzed by organ bath experiments in mouse aortic rings. LPC 18:1 attenuated the acetylcholine-induced, endothelium dependent vasorelaxation and massively decreased NO bioavailability. We conclude that LPC 18:1 induces eNOS uncoupling and unspecific superoxide production. This results in NO scavenging by ROS, a limited endothelial NO bioavailability and impaired vascular function.

## Introduction

Nitric oxide (NO) is a crucial endothelial factor for the maintenance of cardiovascular homeostasis, reflected by its growth regulatory, anti-inflammatory and antithrombotic activities, along with the capacity to promote relaxation of vascular smooth muscle cells and concomitant vasodilation [1], [2]. In vascular endothelium NO is produced by endothelial nitric oxide synthase (eNOS) during conversion of L-arginine to L-citrulline. The activity of eNOS was found to be increased upon binding of Ca^2+^-activated calmodulin and phosphorylation at Ser 1177 [3].

Decreased availability of endothelium-derived NO and increased production of reactive oxygen species (ROS), such as superoxide, hydrogen peroxide or hydroxyl radicals are hallmarks of endothelial dysfunction [4]. Increased cellular superoxide, generated by NADPH oxidase [5], xanthine oxidase [6], cyclooxygenases [7] or mitochondria [8] reacts with NO to form peroxynitrite, a reactive molecule capable of oxidizing the essential cofactor of eNOS, tetrahydrobiopterin (BH4) [9]. This, together with depletion of L-arginine and accumulation of asymmetric dimethyl-L-arginine leads to eNOS uncoupling [1]. Uncoupled eNOS generates superoxide instead of NO, resulting in oxidative stress and NO depletion [10].

Various factors, such as perturbations in blood flow [6] or an altered plasma lipid profile with increased levels of palmitoyl lysophosphatidylcholine (16:0 LPC) modulate vascular NO availability [11]. LPC 16:0 is generated by a variety of reactions including: the cleavage of plasma membrane- and lipoprotein-phosphatidylcholine (PC) by various phospholipase A2 (PLA2) enzymes [12], lecithin cholesterol acyltransferase (LCAT) activity in high-density lipoprotein (HDL) [13], and oxidation of low-density lipoprotein (LDL) [14]. Additional sources of LPC are endothelial lipase (EL) and hepatic lipase (HL), which by cleaving HDL-PC generate substantial amounts of unsaturated oleoyl-LPC (18:1 LPC), linoleoyl-LPC (18:2 LPC) and arachidonoyl-LPC (20:4 LPC), respectively [15], [16]. These LPCs are among the most abundant LPC species in human plasma [17].

The physiological concentration of LPC in plasma is as high as 190 µM [17] with even millimolar levels in hyperlipidemic subjects [18]. Most LPC in plasma is bound to albumin and other carrier proteins and lipoproteins [19], [20]. However minute free LPC might appear in phases of excessive lipolysis and concomitant saturation of albumin and carrier proteins with fatty acids (FA) and LPC, leading to interaction of this free LPC with cells [20]. The vascular function of the mostly studied, saturated LPC 16:0 is discussed controversially: Both has been described: a decrease as well as increase in eNOS synthesis and NO production [21]–[26] and consistently, a promoted or impaired endothelium-dependent relaxation [27]–[29].

In previous studies we found a profound capacity of LPC 18:1 to induce endothelial prostacyclin production [30], interleukin-8 [31] and cyclooxygenase-2 [32] expression as well as potency of attenuating vasorelaxation [29].

In the present study we aimed to examine the impact of LPC 18:1 on NO bioavailability in the human endothelial cell line EA.hy926 [33]. Herein we provide evidence that LPC 18:1 significantly limits the NO bioavailability by augmentation of the cellular oxidative burden.

## Materials and Methods

### Cell culture

Human endothelial cell line EA.hy926 [33] kindly provided by Dr. C.J.S. Edgell (University of North Carolina, Chapel Hill, NC, USA) was cultured in Dulbecco's modified Eagle medium (DMEM) containing 10% fetal bovine serum (FBS) and 1% HAT Media Supplement (all Gibco, Life Technologies). Cell culture medium was supplemented with penicillin G sodium sulfate (100 units/ml), streptomycin sulfate (100 mg/ml), and amphotericin B (2.5 mg/ml) (all Gibco, Life Technologies). Cells were cultured in humidified atmosphere of 5% CO_2_/95% air at 37°C and were sub-cultured using 0.025% trypsin/0.01% EDTA.

### Chemicals

LPC 18:1 (Avanti Polar Lipids) in chloroform was aliquoted under argon, evaporated under nitrogen until dry and stored at −20°C under argon until use. LPC aliquots were dissolved in PBS to yield a stock solution (3 mM) and used fresh for every experiment. NaCl, KCl and CaCl_2_ were from Roth, KH_2_PO_4_ and NaHCO_3_ from Merck (Darmstadt, Germany), MgSO_4_ was from Fluka and α-D-Glucose was from Sigma-Aldrich.

### LPC treatment

#### Treatment of cells

EA.hy926 cells were plated in 12-well dishes 24 h before treatment (120 000/well). Cells were treated with either A) DMEM medium containing 5% FBS supplemented with 60 µM LPC 18:1 (dissolved in PBS) or PBS (vehicle), or B) DMEM medium without FBS, supplemented with 10 µM LPC or PBS (vehicle), for 15 min. Medium was collected for nitrite measurements, and cells were lysed in RIPA buffer (Thermo Fisher Scientific) supplemented with Protease Inhibitor Cocktail from Sigma (1 µl/million cells) and sodium orthovanadate, a phosphatase inhibitor, from Calbiochem (100 µM).

#### Treatment of mouse aortic rings

Rings (2 mm in length) were isolated from the thoracic aorta of 9–12 weeks old male C57BL/6 mice in ice-cold physiological salt solution (PSS) (114 mM NaCl, 4.7 mM KCl, 0.8 mM KH_2_PO_4_, 1.2 mM MgCl_2_, 2.5 mM CaCl_2_, 25 mM NaHCO_3_ and 11 mM D-glucose pH 7.4). Each ring was put into a separate well of a 96-well plate containing 150 µl of PSS followed by incubation in cell culture incubator at 37°C for 1 h. During the last 30 min of 1 h-incubation, some rings were incubated with 100 µM L-NNA. After the1 h-incubation period, PSS was removed from the rings and replaced with fresh warm PSS without FBS containing 10 µM LPC 18:1 or PBS (vehicle) with or without 100 µM L-NNA. After an additional 15-minutes incubation period at 37°C, the PSS was collected and stored at −20°C for subsequent nitrite measurements. The rings were collected and stored at −80°C. The dry weight of rings was measured after lyophilization.

### MTT cell viability assay

3-(4,5-Dimethylthiazol-2-yl)-2,5-Diphenyltetrazolium Bromide (MTT) is reduced by mitochondria of living cells to purple formazan, allowing estimation of cell viability. Cells were incubated with LPC 18:1 or PBS as described above followed by washing with PBS and incubation in FCS free medium containing 0,5 mg/ml MTT for 90 min. Cells were then lysed in 0,04 M HCl in absolute isopropanol, and lysate absorbance was measured in duplicate at wavelengths of 570 and 630 nm. Values measured at 630 nm were used as reference.

### Western blot

Equal amounts of cell lysate protein samples were denatured and subjected to gel electrophoresis using 10% SDS-polyacrylamide gels followed by transfer to PVDF membrane. PeqGOLD protein Marker IV (Peqlab) was used as standard. Proteins were detected by antibodies specific for total eNOS, phosphorylated eNOS (pS1177; pT495) (all BD Transduction Laboratories) and α-tubulin (Cell Signaling), followed by appropriate HRP-conjugated secondary antibodies (Dako). Antibody binding was visualized using Immobilon Western Chemiluminescent HRP Substrate. Densitometric analyses were performed using Image Lab software (Bio Rad).

For the detection of eNOS dimer by Western blot, protein samples were mixed with a 6× SDS loading buffer without β-mercaptoethanol and loaded onto 4–15% gel (BioRad) without denaturation. PeqGOLD protein Marker VII (Peqlab) was used as standard. The electrophoresis was done cold (4°C), followed by transfer to PVDF membrane. The remaining protocol was done as described for total eNOS.

### eNOS activity measurement

Intracellular conversion of L-[^3^H]arginine into L-[^3^H]citrulline was measured as previously described [34]. Briefly, cells grown in 6-well plates were washed and incubated at 37°C with 50 mM Tris buffer, pH 7.4, containing 100 mM NaCl, 5 mM KCl, 1 mM MgCl_2_, 3 mM CaCl_2_, 5% (vol/vol) FBS, L-[2,3-^3^H]arginine (∼10^6^ dpm) and 60 µM LPC 18:1 or PBS (vehicle). Reactions were terminated after 15 min by washing the cells with chilled Tris buffer (50 mM, pH 7.4), containing 100 mM NaCl, 5 mM KCl, 1 mM MgCl_2_ and 0.1 mM EGTA. Subsequent to lysis of the cells with 0.01 N HCl, an aliquot was removed for determination of incorporated radioactivity. To the remaining sample, 200 mM sodium acetate buffer (pH 13.0) containing 10 mM L-citrulline was added (final pH ∼5.0), and L-[^3^H]citrulline separated from L-[^3^H]arginine by cation exchange chromatography.

### ROS measurements

#### Total intracellular ROS

ROS production was measured using 2′,7′-dichlorodihydrofluorescein diacetate (H_2_DCFDA) dye (Biotium, Hayward, CA, USA). Cells grown in 12-well dishes were washed with warm PBS and incubated 20 minutes with 10 µM H_2_DCFDA in PBS (37°C) with or without inhibitors (allopurinol (30 µM), apocynin (100 µM), diethyldithiocarbamic acid diethylammonium salt (DETCA) (20 µM), diphenyliodonium (DPI) (10 µM), N5-[imino(nitroamino)methyl]-L-otnithine (L-NNA) (100 µM), Tiron (100 µM) (all from Sigma), VAS-2870 (10 µM) (Enzo Life Sciences)). The dye was then aspirated and cells were incubated with 60 µM LPC 18:1 or PBS (vehicle) in DMEM medium containing 5% FBS at 37°C for 15 min. Thereafter, medium was aspirated, cells were washed once with cold PBS and lysed with 300 µL 3% (v/v) Triton X-100 in PBS with shaking on ice for 45 min. 50 µL of cold absolute ethanol was then added to the lysate to increase the solubilization of the dye and cells were lysed for additional 15 min. The lysates were then collected and centrifuged (10 min, 13000 rpm, 4°C). Fluorescence was measured in duplicate in white or black 96-well plates at excitation and emission wavelengths of 485 and 540 nm, respectively. Fluorescence was normalized to protein content and fluorescence of PBS (vehicle)-treated cells was set as 1.

#### Superoxide quantification by fluorometry

To measure superoxide production, dihydroethidium (DHE) (Sigma) was used. Cells were plated in 96-well dishes at a density of 20000/well or, in case of microscopy, onto a glass coverslips in a 6-well dish at a density of 250 000/well. 24 hours after plating, cells were washed with warm PBS and incubated for 10 min (37°C) with 15 µM DHE in PBS with the addition of 20 µM DETCA, a superoxide dismutase (SOD) inhibitor. The dye was then aspirated, cells were washed with warm PBS and subsequently incubated with PBS containing 5%FBS, supplemented with 60 µM LPC 18:1 or PBS (vehicle) at 37°C for 15 min. Fluorescence was then measured in a multilabel counter in 96 well plates at excitation and emission wavelengths of 405 and 570 nm, respectively.

#### Superoxide quantification by fluorescent microscopy

For microscopy, cells loaded with DHE were imaged on a digital wide field imaging system, the Till iMIC (Till Photonics Graefelfing, Germany) using a 40× objective (alpha Plan Fluar 40×, Zeiss, Göttingen, Germany), as described recently [35]. For illumination of DHE at 405 nm a monochromator, the Polychrome V (Till Photonics) was used. Emission light was collected at 560 nm. Images were recorded with a charged-coupled device (CCD) camera (AVT Stringray F145B, Allied Vision Technologies, Stadtroda, Germany). For data acquisition and the control of the digital fluorescence microscope the live acquisition software version 2.0.0.12 (Till Photonics) was used. The average intensity of randomly selected single individual cells was extracted using the offline analysis software version 2.0.0.12 from Till Photonics.

### Confocal microscopy

High resolution imaging of subcellular structures was performed in cells loaded with H_2_DCFDA expressing either endoplasmic reticulum-targeted or mitochondria-targeted red fluorescent protein (RFP) after a 15 minute incubation with 60 µM LPC 18:1 or PBS (vehicle). Images were acquired with an array confocal laser scanning microscope, built on an inverse, fully automatic microscope equipped with VoxCell Scan (VisiTech) and a 100× objective (Plan-Fluor 100×/1.45 Oil, Zeiss). H_2_DCFDA was illuminated at 488 nm (120 mW diode laser, Visitron Systems) and emission was collected at 535 nm (ET535/30 m, Chroma Technology Corp.). RFP was excited with 561 nm laser light (50 mW, VSLaserModul, Visitron Systems) and fluorescence was recorded at 630 nm (630/75, Chroma Technology Corp.). Emitted light was acquired with a CCD camera (CoolSNAP-HQ, Photometrics). Background correction and image overlay were performed using the MetaMorph 7.7.0.0 software.

### Amplex Red Assay

The assay was done as described [36]. Briefly, cells were plated in 12-well dishes 24 h before the experiment. Cells were washed once with warm PBS, followed by incubation with 300 µL of pre-warmed assay buffer [HEPES-buffered Tyrode's solution (no FBS) containing 50 µM Amplex Red (Invitrogen) and 2 U/mL Horse Radish Peroxidase (Sigma)] containing 10 µM LPC 18:1 or PBS (vehicle) for 15 min. Polyethylene glycol catalase (PEG-catalase) 300 U/mL or polyethylene glycol superoxide dismutase (PEG-SOD) 75 U/mL (both Sigma) were added in order to detect catalase-sensitive peroxides and superoxide radicals in the medium. The buffer was then transferred to a black 96 well plate and fluorescence was measured at excitation and emission wavelengths of 540 and 580 nm, respectively. Relative fluorescence units were normalised to cellular protein concentration. Final values represent catalase sensitive H_2_O_2_ values obtained by subtration of values measured in the presence of catalase from values obtained upon measurements in the absence of catalse.

### Superoxide determination in mouse aortic rings

Aorta was isolated and cut into rings. Weight of the aortae was recorded for normalization. Rings were incubated with or without 10 µM LPC for 15 minutes followed by 30 minutes of DHE (10 µM) in 37°C. DHE products were extracted from the aortic rings by adding 300 µL acetonitrile. Extracts were concentrated by centrifuging in a Speed Vac machine. Pellets were dissolved in 110 µL of HPLC loading buffer (10% acetonitrile +0.1% TFA in water) and loaded for HPLC measurement [37].

### Nitrite measurements

Nitrite as indicator of NO production was determined according to a previously described fluorometric HPLC method [38]. 100 µL of the cell culture medium or physiological salt solution (PSS) collected following a 15 min LPC –treatment (described above) of cell or aortic rings, were derivatized with 2,3-diaminonaphthalene (DAN) (Sigma-Aldrich, Vienna, Austria). Nitrite thereby reacts with DAN to 2,3-naphthotriazole (NAT). A slight modification to the previously used method [29], consisted of exchange of methanol with acetonitrile in the mobile phase to achieve superior stability and concomitant elution of NAT prior to DAN. To obtain the nitrite values generated by cells or aortic rings, the nitrite values measured in mixtures used to treat cells or aortic rings (DMEM+10% FCS −/+ LPC 18:1, −/+ L-NNA), before their exposure to cells or aortic rings, were subtracted from the nitrite values in those mixtures following incubation with cells or aortic rings. The eNOS specific nitrite values were obtained by subtracting the nitrite values obtained in the presence of 100 µM L-NNA (eNOS inhibitor) from the total measured nitrite in the samples.

### Vascular function studies

Relaxation to cumulatively increasing concentrations of acetylcholine (ACh) were recorded in vessels incubated with LPC 18:1 (10 µM) or PBS (vehicle) for 15 min and preconstricted to 80% of the maximal KCl (60 mmol/L)-induced contraction using norepinephrine (NE), as described [29]. Relaxation values were expressed as a percentage of the initial NE-induced contraction. NO availability was estimated from the constrictor response to the eNOS inhibitor Nω-nitro-L-arginine (L-NA, 300 µM) in aortic rings preconstricted to 10% of the maximal KCl constriction, using phenylephrine in the presence of diclofenac (10 µM) as described [36]. All animals received care in accordance with the Austrian law on experimentation with laboratory animals (last amendment, 2012), which is based on the US National Institutes of Health guidelines. Experiments were approved by the Austrian Federal Ministry for Science and Research (BMWF-66.010/0133-II/3b/2012).

### Statistical analysis

Experiments were performed at least three times and the data are represented as the mean ± standard error of mean (S.E.M.). Differences between groups were assessed using unpaired t-test or Mann-Whitney U test for non-parametric data when comparing two groups, One-way ANOVA with subsequent Tukey's test adjusted for multiple testing for more than two groups, and Two-way ANOVA followed by a Bonferroni post-hoc test for myography experiments (all using Graph Pad Prism 5.0). Statistically significant differences between groups are indicated by *P*-values of <0.05 (*), <0.01 (**), or <0.001 (***).

## Results

### LPC 18:1 limits NO bioavailability in EA.hy926 cells

**Figure 1:**
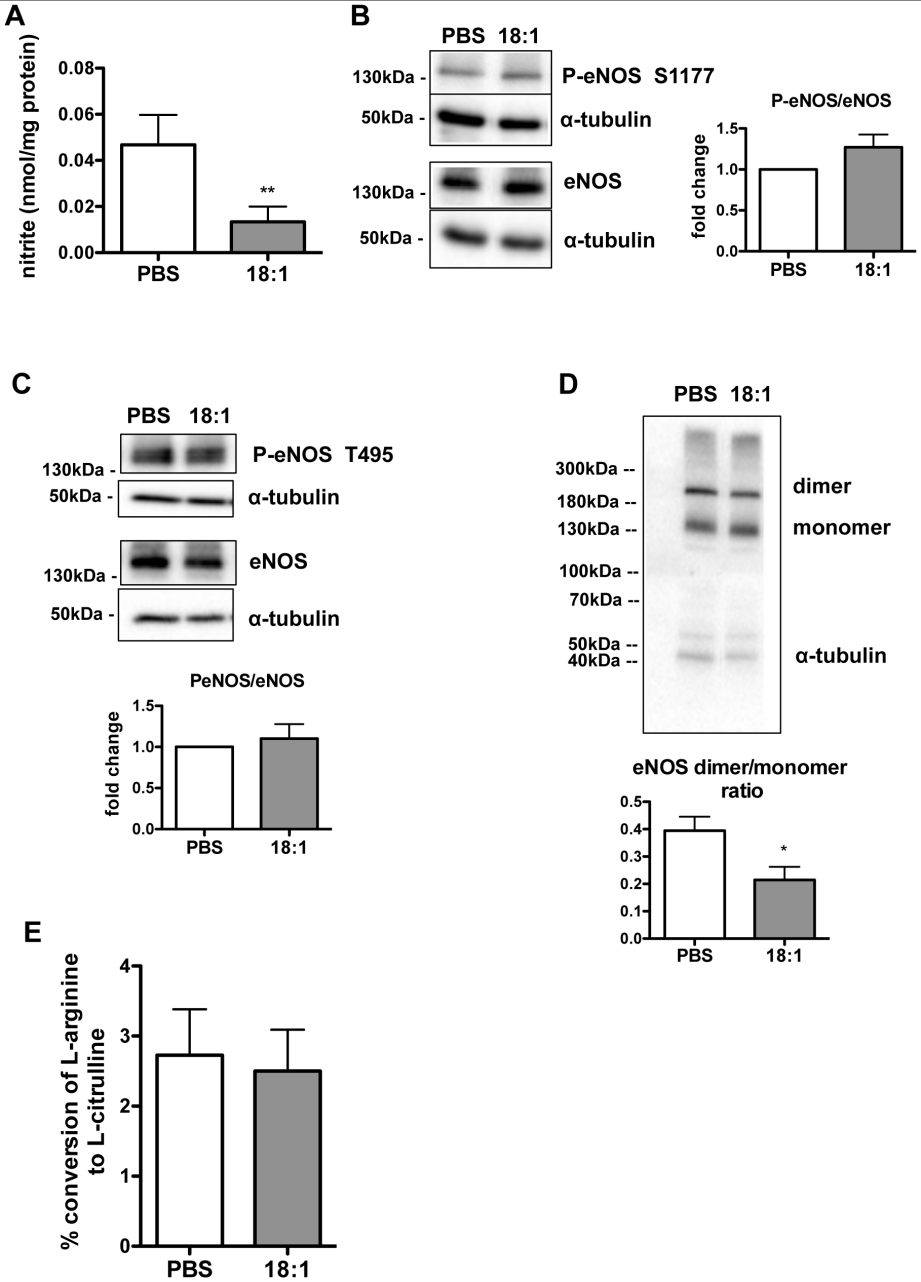
LPC 18:1 lowers NO bioavailability and decreases eNOS dimer in EA.hy926 endothelial cells. Cells were pretreated or not with 100 µM L-NNA for 30 min and then exposed to 60 µM LPC 18:1 or PBS (vehicle) in the presence or absence of 100 µM L-NNA at 37°C in media containing 5% FBS for 15 min. Thereafter cell media were collected for nitrite quantification by HPLC and cell lysates were used for protein content determination. Results show eNOS-dependent nitrite levels obtained by subtraction of values obtained in the presence of L-NNA from values obtained in the absence of L-NNA (A). Cells were incubated with 60 µM LPC 18:1 or PBS (vehicle) as in A followed by Western blot analysis of activating (S1177) (B) or inhibitory (T495) (C) eNOS phosphorylation and eNOS dimer/monomer ratio (D). For all Western blots, protein size annotations refer to protein marker bands on the membrane. (E) eNOS activity was determined in cells exposed to 60 µM LPC 18:1 or PBS (vehicle) along with L-[^3^H]arginine in the presence of 5% FCS for 15 min followed by quantification of L-[^3^H]citrulline after its separation from L-[^3^H]arginine by exchange chromatography. The values are mean ± SEM, of at least 3 independent experiments performed in triplicates and analyzed by Mann-Whitney U test (A,E) or unpaired t-test (B,C,D); *p<0,05,**p<0,01.

To examine the impact of acute, short exposure of cells to LPC 18:1 on NO bioavailability, cells were incubated with this LPC or PBS (vehicle) at concentration of 60 µM in the presence of 5% FBS for 15 min. The viability of cells was not significantly altered by LPC (Figure S1). The nitrite quantification in cell media by HPLC revealed that LPC 18:1 led to a significant decrease in nitrite levels, compared with control PBS-treated cells (Figure 1 A). To examine the underlying mechanism responsible for the decreased nitrite levels in LPC 18:1-treated cells, we analyzed the phosphorylation status of eNOS. Neither the activating phosphorylation on Ser1177 (Figure 1 B) nor the inhibitory phosphorylation on Thr495 (Figure 1 C) were altered by LPC 18:1. However, importantly, the eNOS dimer to monomer ratio (Figure 1 D) was significantly decreased in LPC 18:1- compared with PBS- treated control cells. The decreased abundance of the eNOS dimer was accompanied with a slight, not significantly decreased eNOS activity in LPC 18:1- compared with PBS-treated control cells (Figure 1 E).

### LPC 18:1 augments intracellular ROS in EA.hy 926 cells

**Figure 2:**
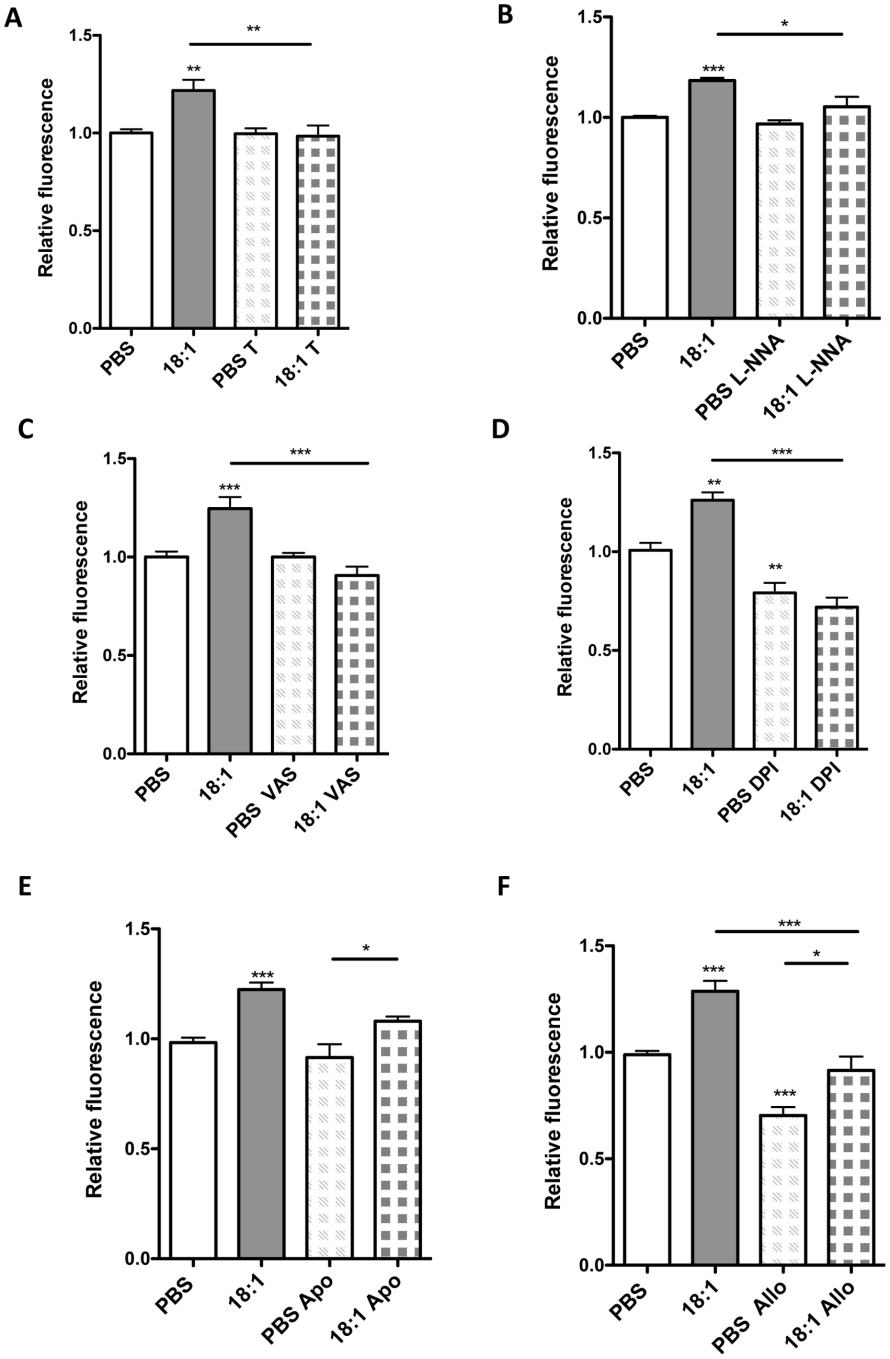
LPC 18:1 increases intracellular ROS formation. Cells were incubated with 10 µM H_2_DCFDA dye in PBS in the absence or presence of (A) 100 µM Tiron (T), (B) 100 µM L-NNA, (C) 10 µM VAS2870 (VAS), (D) 10 µM DPI (E) 100 µM apocynin or (F) 30 µM allopurinol for 20 min, followed by exposure to 60 µM LPC 18:1 or PBS (vehicle) in the absence or presence of inhibitors, in medium containing 5% FBS at 37°C for 15 min. Results are mean ± SEM of 3 independent experiments done in triplicates, and analyzed by one-way ANOVA with Tukey's post-hoc test. If otherwise not indicated, asterisks show significance compared to PBS control; *p<0,05, **p<0,01, ***p<0,001.

Because the decreased NO bioavailability and eNOS dimer to monomer ratio might be a consequence of increased oxidative stress [1] we assumed increased ROS levels in cells exposed to LPC 18:1. Indeed, 60 µM LPC 18:1 applied in the presence of 5% FBS led to increased ROS levels. This increase in ROS could be prevented by preincubation of cells with the cell permeable superoxide scavenger Tiron (Figure 2 A). To examine the relative contribution of various cellular enzymes to LPC 18:1-mediated increase in ROS levels cells were exposed to 60 µM LPC 18:1 in the presence of 5% FBS or PBS (vehicle) in the absence or presence of corresponding pharmacological inhibitors. While eNOS inhibitor L-NNA (Figure 2 B) and NADPH oxidase inhibitors VAS-2870 [39] (Figure 2 C) significantly decreased LPC 18:1-induced ROS, the inhibition of all flavine-containing enzymes by DPI decreased both basal and LPC 18:1-induced ROS (Figure 2 D). The attenuating effect of apocynin (NADPH oxidase inhibitor and antioxidant [40]) was weak and did not reach statistical significance (Figure 2 E). The xanthine oxidase inhibitor allopurinol decreased ROS levels in both PBS and LPC 18:1-treated cells, but failed to significantly alter the LPC 18:1-induced ROS formation (Figure 2 F).

### LPC 18:1-induced ROS is localized in cytosol and mitochondria

**Figure 3:**
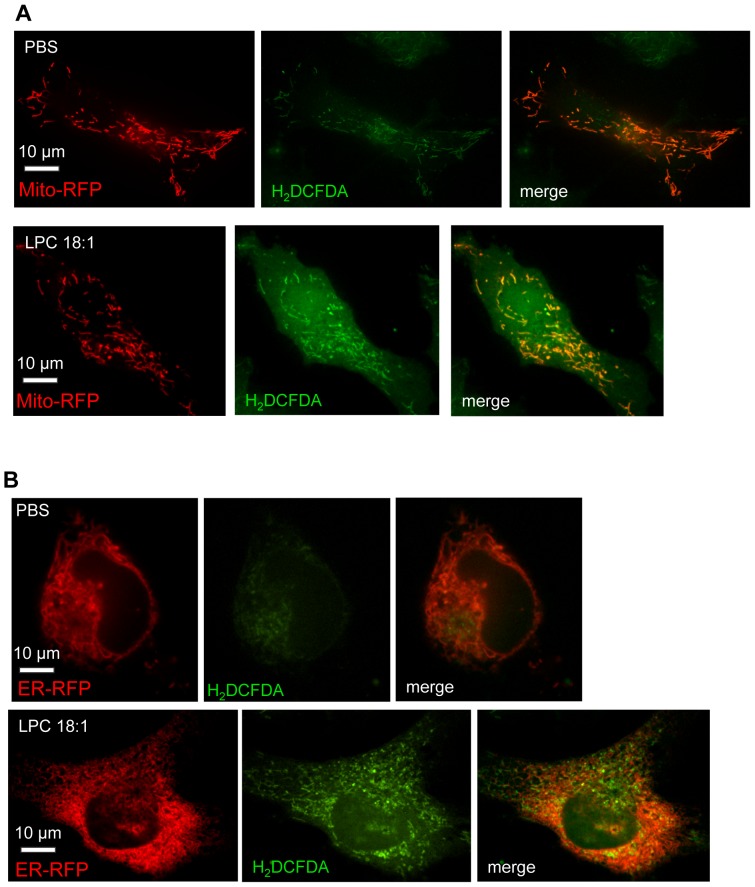
LPC 18:1 induced ROS localizes mainly in mitochondria and cytosol. Twenty four hours after transfection of EA.hy926 cells with plasmids encoding either (A) mitochondria- or (B) ER- targeted RFP, cells were labeled with H_2_DCFDA dye and exposed to 60 µM LPC 18:1 or PBS (vehicle) in PBS containing 5% FBS at 37°C for 15 min. Fluorescence was assessed by confocal microscopy using mitochondrial- and endoplasmic reticulum-specific RFP marker (red) and a H_2_DCFDA ROS marker (green). Colocalization (yellow) was achieved by merging RFP and ROS signals (merge). Results are representative images of two experiments performed in triplicates.

The involvement of various enzymes in LPC 18:1-induced ROS production (Figure 2) prompted us to examine subcellular localization of ROS in cells exposed to 60 µM LPC 18:1 in the presence of 5% FBS and control cells exposed to PBS (vehicle), expressing mitochondria-targeted RFP (Mito-RFP) or endoplasmic reticulum-targeted RFP (ER-RFP) by fluorescent microscopy. The ROS signal (H_2_DCFDA) was more pronounced in LPC 18:1 compared with control cells (Figure 3 A,B). Merged images showed profound but not complete colocalization (yellow) of the H_2_DCFDA signal with Mito-RFP (Figure 3 A) and no colocalization of the H_2_DCFDA signal with ER-RFP (Figure 3 B).

### LPC 18:1 increases intracellular and extracellular superoxide levels

**Figure 4:**
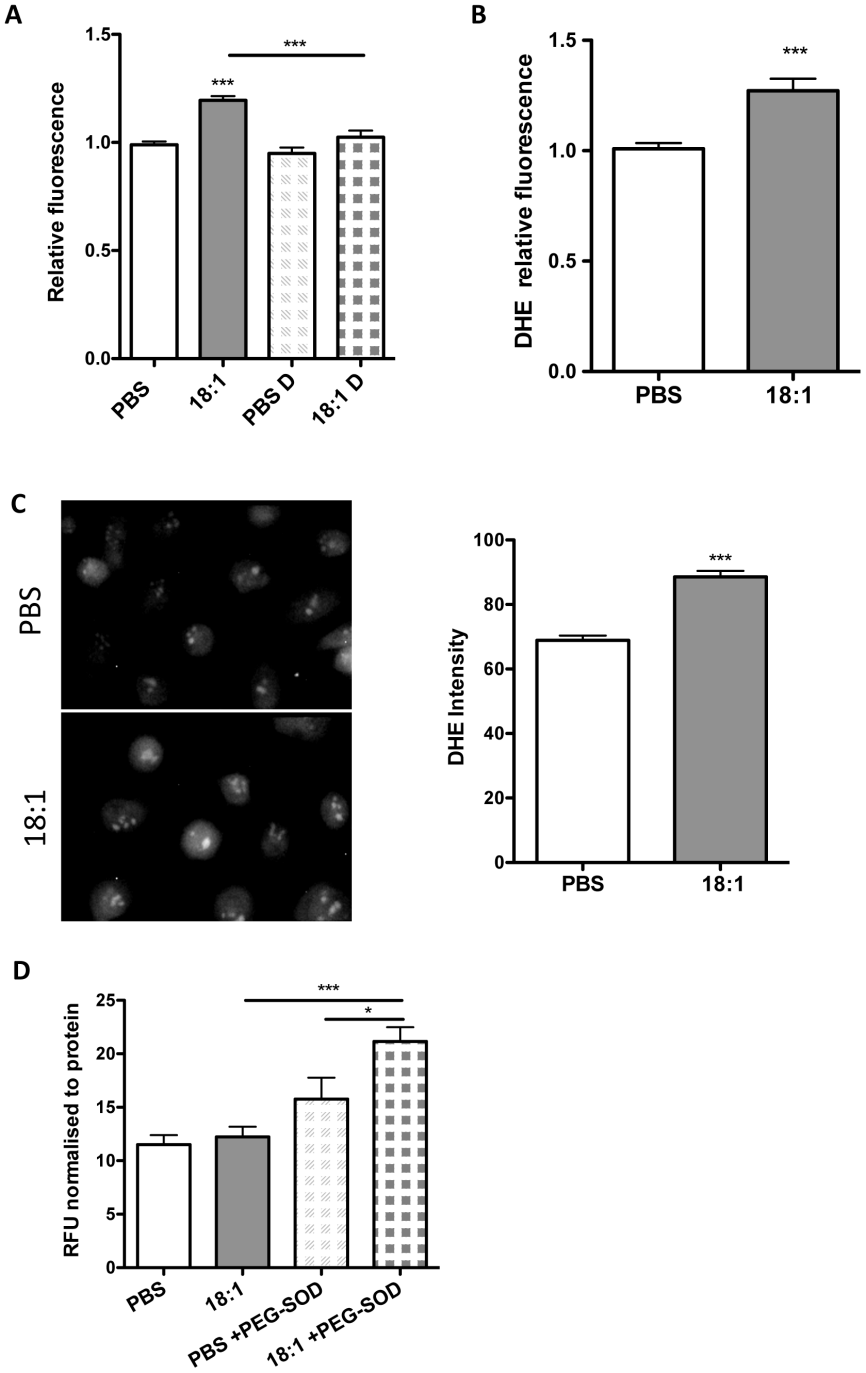
LPC 18:1 induces intracellular and extracellular superoxide production. (A) ROS levels were measured as in Figure 2 after inhibition of SOD with 20 µM DETCA. (B) Cells plated in 96-well dishes were incubated with 15 µM DHE in the presence of 20 µM DETCA at 37°C for 15 min. Thereafter, cells were exposed to 60 µM LPC 18:1 or PBS (vehicle) in PBS containing 5% FBS at 37°C for 15 min, followed by fluorometric superoxide quantification. (C) Cells grown on a glass coverslip were treated as in B followed by fluorescent microscopy. (D) Amplex Red Assay was performed in cells exposed to 10 µM LPC 18:1 or PBS in the presence or absence of PEG-catalase (300 U/ml) or PEG-SOD (75 U/ml) in HEPES-buffered Tyrode's solution without FBS at 37°C for 15 min. The presented values are catalase-sensitive values obtained by subtraction of values obtained in the presence of catalase from those in the absence of catalase. The values shown are mean ± SEM of 3 independent experiments performed in triplicates and analyzed by one-way ANOVA with Tukey's post-hoc test (A,D) or unpaired t-test (B,C). If otherwise not indicated, asterisks show significance compared to PBS control; *p<0,05, ***p<0,001.

To examine the contribution of LPC 18:1-induced superoxide to the total ROS identified with H_2_DCFDA, the total ROS levels were measured in cells exposed to 60 µM LPC in the presence of 5% FBS, upon inhibition of SOD by DETCA. Almost complete inhibition of LPC 18:1-induced increase in the total ROS levels measured by H_2_DCFDA (Figure 4 A) strongly indicated induction of superoxide in LPC 18:1-treated cells. Indeed, in cells labeled with DHE, a dye specific for superoxide, we identified by both fluorometry (Figure 4 B) and fluorescent microscopy (Figure 4 C) increased signal (superoxide levels) in LPC 18:1- compared with PBS-treated control cells. To examine whether extracellular ROS levels were altered by LPC 18:1, the ROS levels in cell media were measured using Amplex Red, a dye specific for hydrogen peroxide. For this assay cells were exposed to 10 µM LPC 18:1 in media without FBS or to PBS (vehicle) in the presence or absence of PEG-SOD. An increased ROS signal only in cells exposed to LPC 18:1 in the presence of PEG-SOD but not in the absence of PEG-SOD or in PBS-treated cells (Figure 4 D), demonstrated the LPC 18:1-induced increase in extracellular superoxide.

### LPC 18:1 limits NO bioavailability in cells and mouse aortic segments

**Figure 5:**
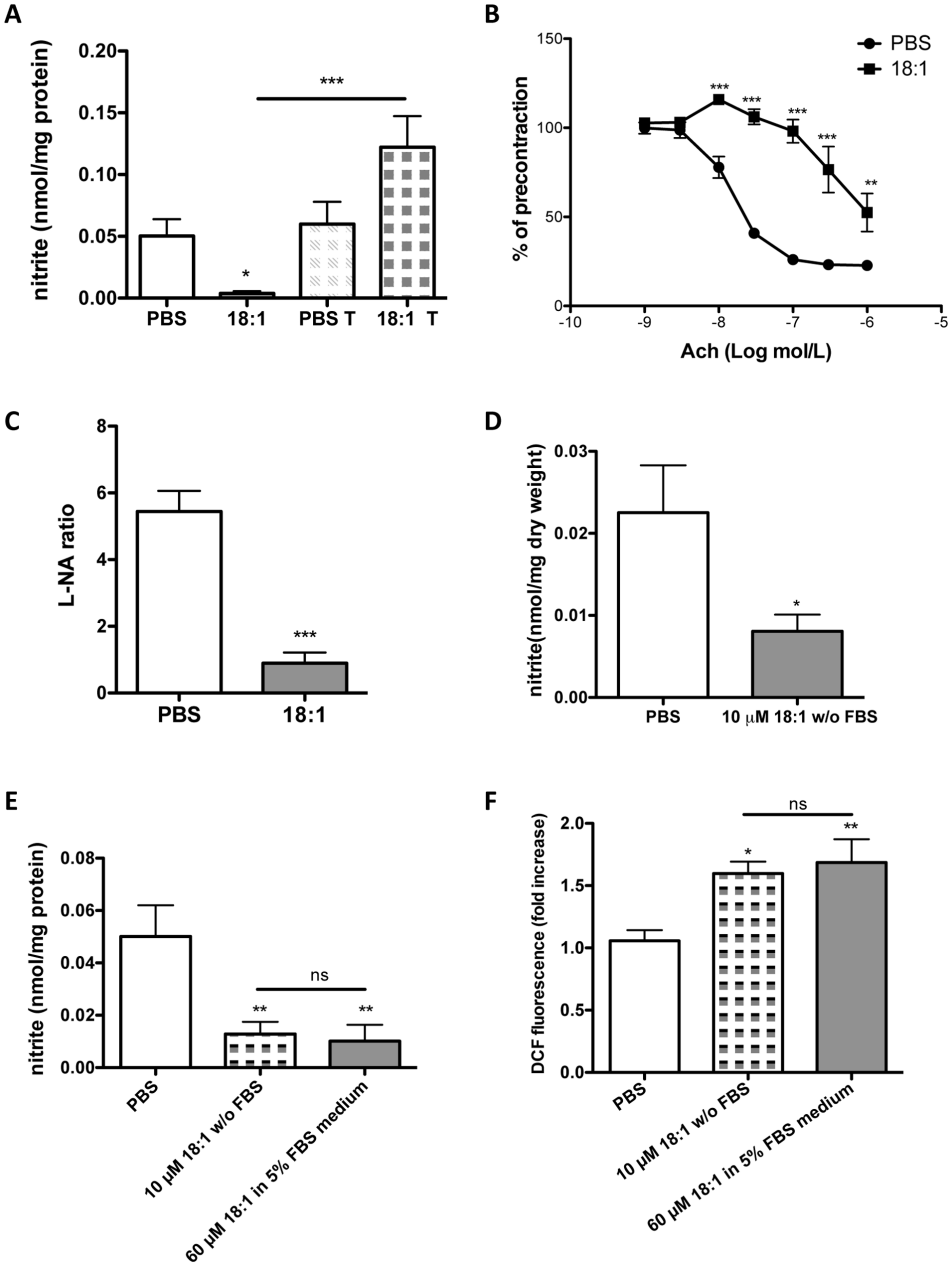
LPC 18:1 limits NO bioavailability in cells and mouse aortic segments. (A) Nitrite levels in cell media of LPC 18:1- or PBS-treated cells were measured as in Figure 1 A in the absence or presence of 100 µM Tiron. (B) Mouse aortic rings (5 for each condition) were incubated with 10 µM LPC 18:1 or PBS in the absence of FBS for 15 min followed by NE preconstriction and relaxation to cumulative addition of Ach. Relaxation values are expressed as a percentage of the initial NE-induced contraction. (C) Mouse aortic rings were treated with 10 µM LPC 18:1 or PBS in the absence of FBS for 15 min and preconstricted with phenylephrine to 10% of maximal contraction followed by addition of 300 µM L-NA (eNOS inhibitor). The ratio (L-NA ratio) of constriction achieved after and before addition of L-NA is shown. (D) Mouse aortic rings were treated with 10 µM LPC 18:1 or PBS in PSS without FBS for 15 min. Nitrite levels determined in the buffer (PSS) post incubation are shown. (E) Cells were treated with 10 µM LPC 18:1 (in medium without FBS) or with 60 µM LPC 18:1 or PBS (both in medium with 5% FBS) for 15 minutes. Nitrite levels determined in the media after treatment are shown. (F) ROS species were measured with H_2_DCFDA dye (as in Figure 2) after exposure of cells to 10 µM LPC 18:1 (in medium without FBS) or to 60 µM LPC 18:1 or PBS (both in medium with 5% FBS) for 15 minutes. Results are mean ±SEM of 3 independent experiments done in triplicate and analyzed by one-way ANOVA with Tukey's post-hoc test (A, E, F) or by two-way ANOVA with Bonferroni post-hoc test (B) or unpaired t-test (C, D). If otherwise not indicated, asterisks show significance compared to PBS control; *p<0,05, **p<0,01, ***p<0,001, ns –not significant.

Considering LPC 18:1-induced oxidative burden as a major cause for decreased NO bioavailability, we tested the capacity of Tiron, capable of preventing LPC 18:1-mediated increase in ROS (Figure 2 A), to recover NO bioavailability in LPC 18:1-treated EA.hy926 cells. As shown in Figure 5 A a marked decrease in NO (nitrite) in cells exposed to 60 µM LPC 18:1 in the presence of 5% FBS could be completely circumvented by Tiron. To further study the impact of LPC 18:1 on NO bioavailability we performed bioassay experiments in mouse aortic rings. First, we demonstrated that 10 µM LPC 18:1 in the absence of FBS caused a marked impairment of Ach-induced relaxation of NE-precontracted aortic rings (Figure 5 B). As a second measure for NO bioavailability, we determined the L-NA-induced endothelium-dependent constrictor response in phenylephrine-preconstricted aortic rings. This constrictor response was markedly impaired in rings exposed to 10 µM LPC 18:1 in the absence of FBS compared with PBS-treated control rings (Figure 5 C). Additionally, we observed decreased NO (nitrite) in supernatants of aortic rings treated with 10 µM LPC 18:1 in the absence of FBS compared with control PBS incubations (Figure 5 D). The increase in superoxide levels in LPC 18:1-treated rings showed an increasing trend, however, without reaching statistical significance (Figure S2.). Because experimental conditions in cell culture experiments (except experiments shown in Figure 4 D) and in aortic rings differed in terms of LPC concentrations and the presence of FBS, we compared the impact of 10 µM LPC 18:1 applied without FBS with 60 µM LPC 18:1 applied in the presence of 5% FBS on NO bioavailability and ROS formation in EA.hy926 cells. Both experimental conditions caused a similar LPC 18:1-induced decrease in NO bioavailability (Figure 5 E) as well as increase in ROS production (Figure 5 F).

## Discussion

We previously reported impact of LPC 18:1 on endothelial prostacyclin production [30], interleukin-8 [31] and cyclooxygenase-2 [32] expression as well as vasorelaxation [29]. In the present study we examined acute effect of this LPC on the endothelial NO bioavailability, a hallmark of endothelial health. EA.hy926 cells were shortly exposed to 60 µM LPC (15 min) in the presence of 5% FBS. The applied LPC concentration in combination with 5% serum corresponds to 1.2 mM LPC in 100% serum. While 25 µM LPC 18:1 plasma levels (13% of 190 µM total LPC) are found under physiological conditions [17], 1.2 mM LPC 18:1 levels are likely under pathophysiological conditions. For example it has been shown that hyperlipidemic subjects have millimolar LPC levels [18]. Furthermore, a transient increase in LPC 18:1 levels in vivo is conceivable during acute inflammatory response and concomitantly excessive lipolysis catalyzed by EL on the surface of vascular endothelium [15], [41]. Under such conditions LPC concentrations might exceed the binding capacity of albumin, known to attenuate interaction of LPC with cells [30], [42]. Additionally, LPC scavenging by albumin might be impaired by free fatty acids [17] excessively produced by EL under inflammatory conditions [15], [41], [43]. Moreover, a combination of inflammatory state and conditions of decreased albumin levels, as encountered in patients with renal failure on hemodialysis [44], may by increasing both the absolute and relative abundance of LPC, promote detrimental effects of LPCs including LPC 18:1 on endothelium. Based on the fact that the critical micellar concentration for LPC 16:0 was estimated to be between 7 and 50 µM (depending on the conditions like temperature, salt concentration, pH or presence of proteins or lipids [45]–[47]) one can only speculate whether free LPC under given experimental conditions exist as single molecules or micelles. Previous studies reported both decrease and increase in eNOS levels and NO bioavailability in cells exposed to LPC 16:0 for periods between 2 and 24 h [21]–[26]. In contrast to these studies, the effects of LPC 18:1 on NO bioavailability in our experimental model comprise exclusively rapid molecular events, independent of induction of mRNA or protein synthesis.

Previous studies on the impact of LPC species on endothelial NO bioavailability used exclusively LPC 16:0 but not unsaturated LPC species. To the best of our knowledge this is the first study addressing the impact of unsaturated LPC on NO bioavailability in endothelial cells. We found that LPC 18:1 significantly limited NO bioavailability in endothelial cells. This finding was accompanied with partial disruption of the active eNOS dimer, accompanied by eNOS uncoupling and only slightly, not significantly decreased eNOS activity. Considering the established impact of oxidative burden on eNOS [1], the disruption of eNOS dimer and eNOS uncoupling result in an increased ROS formation. It remains to be determined whether oxidation of the critical eNOS cofactor tetrahydrobiopterin, or local depletion of eNOS substrate L-arginine per se or via acute induction of arginases caused eNOS uncoupling in cells exposed to LPC 18:1 [1]. Although accumulation of an endogenous eNOS inhibitor, asymmetric dimethylarginine (ADMA) was observed in endothelial cells exposed to LPC 16:0 for 24 h [25], it is unlikely that in our experimental model a 15 min- exposure of cells to LPC 18:1 is sufficient to allow accumulation of ADMA. Recently we found that a short incubation with LPC results in a significantly increased LPC levels in endothelial cells [41]. This is in line with a very rapid incorporation of LPC into the plasma membrane [20], [41], [48], [49]. Accordingly, it is tempting to speculate that LPC 18:1 taken up by cells interacts directly with eNOS thus changing structural integrity and functionality of the enzyme. Additionally, LPC may by incorporation into the cell membrane change the bilayer thickness and leaflet curvature balance [50] or change the composition and stability of caveolae leading to altered interaction of eNOS with caveolin-1 or heat shock protein 90, both involved in modulation of eNOS integrity and activity [51]. The observed weak and non-significant decrease in eNOS activity could not explain a profound decrease in NO bioavailability, strongly arguing for the role of LPC 18:1 induced ROS in NO degradation.

A consequence of eNOS uncoupling is an increased and uncontrolled ROS formation. In the present study a short exposure of cells to LPC 18:1 significantly increased intracellular and extracellular ROS levels, mediated by various enzymes from different subcellular compartments. From ROS measurements in the presence of pharmacological inhibitors of ROS-producing enzymes and from fluorescent microscopy data, it appears that NADPH oxidases, uncoupled eNOS and additional flavoprotein-containing enzymes in cytosol and mitochondria are major sources of ROS in LPC 18:1-treated cells. A strong ROS signal in mitochondria of LPC 18:1-treated cells is consistent with a previous report showing LPC 16:0-mediated induction of mitochondrial ROS in human umbilical vein endothelial cells [52]. Several previous studies demonstrated ROS induction in cells exposed to LPC 16:0, with the argumentation based on the inhibitory effects of DPI, for the role of NADPH oxidases in ROS generation [17], [25], [53]–[55]. Considering the inhibitory effect of DPI on many different flavoprotein-containing enzymes it is likely that many different enzymes in addition to NADPH oxidase, including those in mitochondria as well as uncoupled eNOS, like in our study, contributed to LPC induced ROS formation in those studies. However, the exact intracellular localization and the relative quantitative as well as temporal contributions of the various ROS sources in LPC 18:1-treated cells require further investigation.

In addition to increased intracellular ROS we observed increased superoxide levels in cell media of LPC 18:1-treated cells. Because the plasma membrane is poorly permeable for superoxide anions their increase in extracellular compartment most likely reflects contribution of plasma membrane localized uncoupled eNOS. The uncoupling of eNOS results in an enhanced ROS-formation by eNOS itself, which in turn may trigger ROS production by a variety of sources [56]. The augmented production of superoxide anions further limits NO bioavailability by the formation of peroxynitrite. As a consequence, vasorelaxation is strongly impaired in the presence of LPC 18:1. Because serum interferes with myography measurements due to extensive foam formation and because 60 µM LPC 18:1 would under serum-free conditions be detrimental to aortic rings, the impact of LPC 18:1 on vascular function was studied in aortic rings exposed to 10 µM LPC 18:1 in the absence of serum for 15 min. Such free LPC might appear in vivo during excessive lipolysis when albumin and other carrier plasma proteins are saturated with FA and LPC [20]. We found previously that 10 µM LPC 18:1 when applied in the absence of serum is not toxic to cultured endothelial cells or aortic rings [29], [30]. In the present study we show that 10 µM LPC 18:1 applied in the absence of serum impacts NO bioavailability and ROS production similarly to 60 µM LPC applied with 5% FBS.

Since increased oxidative burden is the major player responsible for the attenuation of endothelium-dependent relaxation in aged vessels and in various pathologies (essential hypertension, diabetes, dyslipidemia and atherosclerosis) [4] the augmentation of ROS with concomitantly decreased NO in vascular endothelium exposed to LPC 18:1 strongly argues for the conceivable contribution of this LPC to endothelial dysfunction in aging and aforementioned pathologies. The relevance of our data obtained in cell culture and aortic rings for human cardiovascular pathophysiology is highlighted by a most recent report on increased LPC 18:1 plasma levels in prehypertensive patients compared with normotensive controls [57].

## Conclusions

Based on the results of the present study we conclude that eNOS uncoupling and augmented ROS production with concomitant NO scavenging cause the reduction of NO bioavailability in endothelial cells and mouse aortic rings exposed to LPC 18:1.

## Supporting Information

Figure S1 LPC 18:1 has no effect on cell viability. MTT test (MTT reduction to formazan), was performed following exposure of cells to 60 µM LPC 18:1 or PBS in media containing 5% FBS at 37°C for 15 min. Results are mean ± SEM of 3 independent experiments performed in triplicates and analyzed by unpaired t-test.
(TIF)

Figure S2 Superoxide levels in mouse aortic segments exposed to LPC 18:1. Mouse aortic segments were incubated with 10 µM LPC 18:1 or PBS in the absence of FBS, followed by 30 min incubation with superoxide- specific DHE (10 µM), after which the oxidized products were extracted and measured by HPLC. Results are mean ± SEM of measurements in aortic segments of 3 animals per condition, analyzed by unpaired t-test.
(TIF)
