# Supplementary figures and images for: Oleoyl-Lysophosphatidylcholine Limits Endothelial Nitric Oxide Bioavailability by Induction of Reactive Oxygen Species
Source: PLoS One. 2014 Nov 24;9(11):e113443. doi: 10.1371/journal.pone.0113443 (PMC4242637; doi:10.1371/journal.pone.0113443)

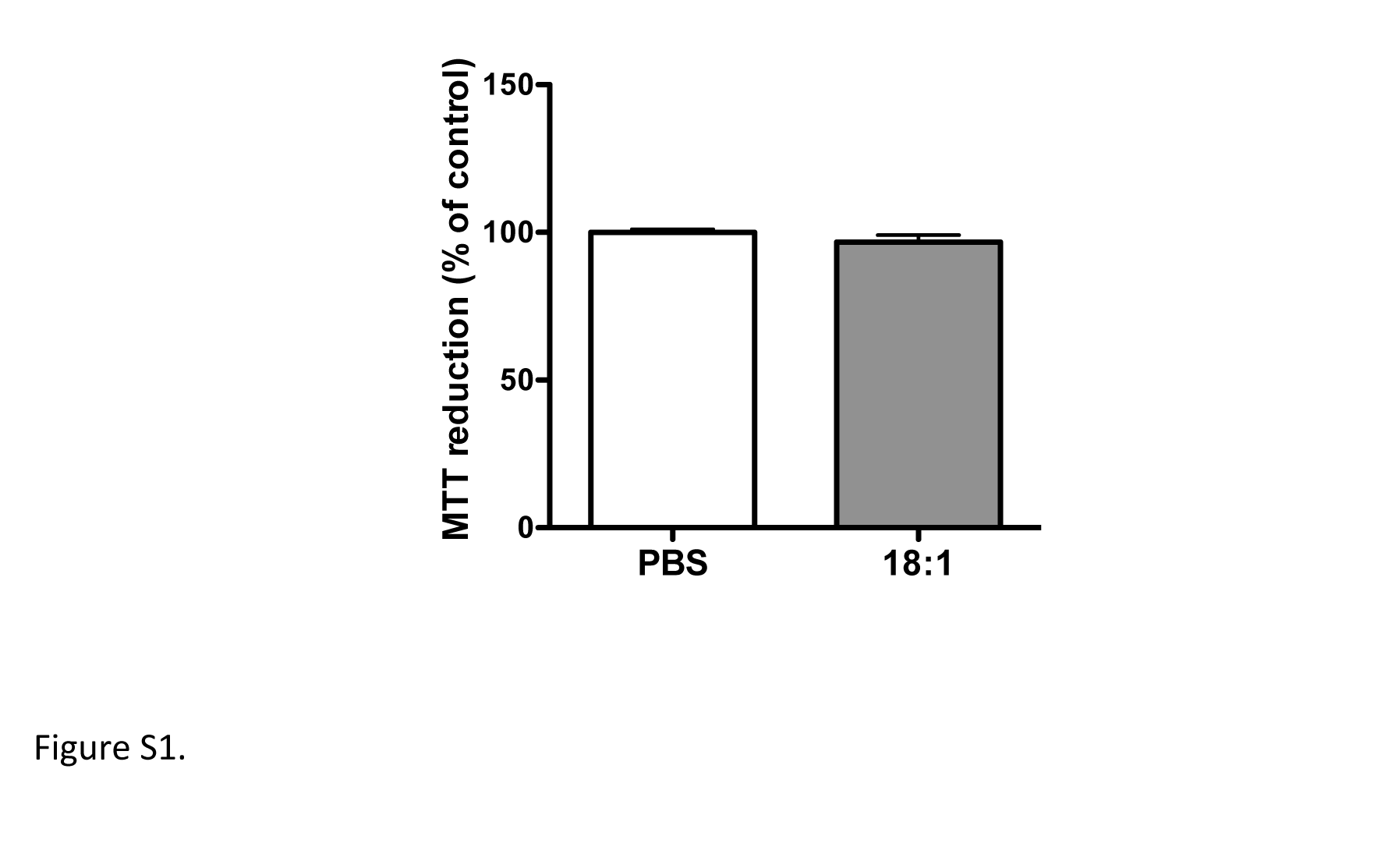

Supplement: Figure S1 — LPC 18:1 has no effect on cell viability. MTT test (MTT reduction to formazan), was performed following exposure of cells to 60 µM LPC 18:1 or PBS in media containing 5% FBS at 37°C for 15 min. Results are mean ± SEM of 3 independent experiments performed in triplicates and analyzed by unpaired t-test. (TIF) [file pone.0113443.s001.tif]

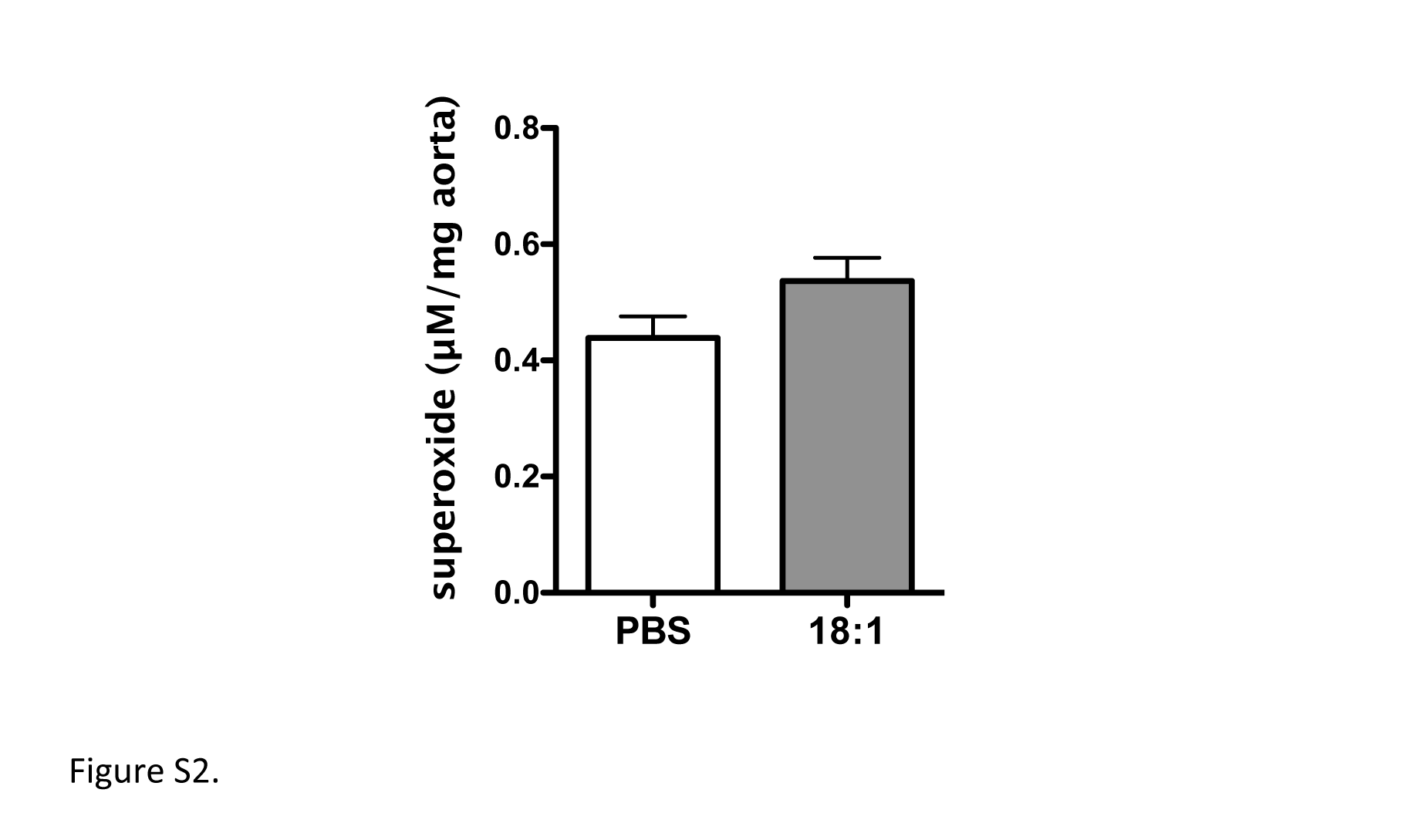

Supplement: Figure S2 — Superoxide levels in mouse aortic segments exposed to LPC 18:1. Mouse aortic segments were incubated with 10 µM LPC 18:1 or PBS in the absence of FBS, followed by 30 min incubation with superoxide- specific DHE (10 µM), after which the oxidized products were extracted and measured by HPLC. Results are mean ± SEM of measurements in aortic segments of 3 animals per condition, analyzed by unpaired t-test. (TIF) [file pone.0113443.s002.tif]
